# Development of Monoclonal Antibodies and Immunoassays for Sensitive and Specific Detection of Shiga Toxin Stx2f

**DOI:** 10.1371/journal.pone.0076563

**Published:** 2013-09-17

**Authors:** Craig Skinner, Stephanie Patfield, Larry Stanker, Xiaohua He

**Affiliations:** Western Regional Research Center, United States Department of Agriculture, Agricultural Research Service, Albany, California, United States of America; Wadsworth Center, New York State Dept. Health, United States of America

## Abstract

**Background:**

Shiga toxin *2* (Stx2) is a major virulence factor in gastrointestinal diseases caused by *Escherichia coli*. Although Stx2a (prototypical Stx2) is well-studied, all seven subtypes of Stx2 have been associated with disease in mammals. Several subtypes of Stx2, including Stx2f, are difficult to detect immunologically.

**Methods And Findings:**

Four novel monoclonal antibodies (mAbs) against the Stx2f subtype were produced and characterized. These mAbs react exclusively to the Stx2f A subunit, and do not cross-react with other subtypes of Stx2. A Stx2f-specific sandwich ELISA was established and a limit of detection of 0.123 ng/mL was obtained using one pair of the mAbs. The receptor preference of Stx2f was confirmed using this sandwich ELISA. Three out of four mAbs can partially neutralize the toxicity of Stx2f in a cell-based assay. These mAbs were also demonstrated to be highly specific and reactive when applied to colony immunoblot assays.

**Conclusions:**

Novel mAbs specific to Stx2f were developed for the first time, providing new assets for the STEC community. Immunoassays with improved sensitivity and specificity will be useful for the detection of Stx2f present in food, environmental, and clinical samples.

## Introduction

Food poisoning of bacterial origins is widespread and a considerable public health concern both in the US and worldwide. In the US alone, the Centers for Disease Control and Prevention estimates that 9.4 million Americans contract foodborne illness each year [1]. Foodborne bacterial contaminants such as *Salmonella spp.*, *Listeria* *spp.* , *Campylobacter* *spp.* , and *Escherichia coli* (*E. coli*) account for a considerable portion of these cases, and these four pathogens are responsible for many of the most deadly outbreaks [2,3]. *E. coli* strains expressing Shiga toxin (Stx), known as STEC (Shiga toxin-producing *E. coli*), can result in a wide variety of clinical manifestations, ranging in severity from innocuous diarrhea to hemorrhagic colitis and life-threatening hemolytic uremic syndrome (HUS) [4]. Phage encoded Stx is among the most important virulence factors for enterohaemorrhagic *E. coli* (EHEC) [5,6] and enteroaggregative hemorrhagic *E. coli* (EAHEC) [7,8]. Many serotypes of EHEC, a class of pathogenic *E. coli* that can cause bloody diarrhea, possess one or several *stx* genes. Enteroaggregative *E. coli* (EAEC) are characterized by their ability to attach to cells which line the intestine; EAHEC additionally have the ability to cause bloody diarrhea [9]. The O104:H4 strain of *E. coli* responsible for the most deadly recent outbreak of STEC in Germany (2011) is classified as EHAEC (or EAEC) and possesses a *stx* gene (*stx2*) [10].

The diversity of STEC strains, both in the genes repertoires they possess and the virulence factors they encode, is considerable. Although the *E. coli* O157:H7 serotype is the most infamous, non-O157 serotypes are responsible for a considerable number of STEC outbreaks. Six O groups (O26, O45, O111, O121, O103, and O145) cause approximately 71% of non-O157 outbreaks [11]. The EAEC strain O104:H4 caused one of the worst *E. coli* incidents in history, a mass outbreak of STEC in Germany in 2011, affecting 3816 people and resulting in 845 cases of HUS and 54 deaths [7,8,12]. These serotypes can harbor one or more *stx* genes, of which there are many varieties. These genes are carried by lambdoid bacteriophages, which can facilitate the transfer of *stx* sequences between STEC serotypes, non-pathogenic *E. coli* [13], and possibly other close relatives to *E. coli* in Enterobactericiae [14,15]. The two main types of Stx include Stx1, which is nearly identical to the toxin from the *Shigella* genus, and Stx2, which is considerably different from Stx1 (only 56.6% amino acid identity between A subunits without signal sequences).

Like several other bacterial toxins, Stx has an AB_5_ structure: the catalytic A subunit is delivered to target cells by a B subunit pentamer. The B subunit pentamer binds the glycolipid receptors globotriaosylceramide (Gb3Cer) and/or globotetraosylceramide (Gb4Cer) on the surface of target cells, allowing entry of the A subunit which then inactivates ribosomes via its *N*-glycosidase activity [16,17]. Although Stx1 seems to be more toxic to Vero cells [18], Stx2 is the much more potent toxin *in vivo*: Stx2 is around 100 times more toxic to mice than Stx1. Stx2 seems to have comparable catalytic activity to Stx1 [19]. The A and B subunits of Stx1 and Stx2 possess N-terminal signal sequences which facilitate their transport to the periplasm, where they assemble into mature toxin [20,21]. Expression of Stx is driven by a late-phase phage promoter, which is strongly activated upon induction of the bacterial SOS response. Expression of Stx1 is additionally dependent upon a bacterial promoter that is responsive to iron concentration [22]. The SOS response also initiates lysis of *E. coli* cells by the phage, resulting in release of the toxin. Some antibiotics, such as the quinolones (e.g., ciprofloxacin), exacerbate the effects of Stx toxicity, presumably by inducing and releasing large amounts of toxin at once [23,24]. Treatment of STEC by these antibiotics might actually worsen the symptoms of STEC infections [25]. Because of this, there are currently no widely accepted antibiotic treatments of STEC infections, although proper antibiotic treatment may ultimately improve the prognosis of patients with the potentially life-threatening HUS [26].

Within each Stx type (Stx1 and Stx2), there are a number of subtypes which vary in sequence, specificity, and toxicity. There are 3 characterized subtypes of Stx1 (Stx1a, Stx1c, and Stx1d) and 7 subtypes of Stx2 (Stx2a, 2b, 2c, 2d, 2e, 2f, and 2g) [27]. The subtypes of Stx1 are relatively conserved at the amino acid level, whereas those of Stx2 can be more diverse. However, the Stx2a, Stx2c, and Stx2d subtypes are very similar to each other, and these subtypes are typically associated with HUS [18,28]. Stx2b, Stx2e, Stx2f, and Stx2g are less commonly found in serious human disease, although Stx2e can cause edema disease in neonatal piglets [29]. Stx2f (found mostly in avian isolates) [30] is the most unique of the Stx2 subtypes (73.9% identity to Stx2a in the A subunits), followed by 2b (93.3%), Stx2e (93.9%), and finally Stx2g (94.9%). Differences among the B subunits determine each subtype’s receptor specificity. Stx2a, Stx2c, and Stx2d bind preferentially to Gb3Cer, while it has been reported that Stx2e prefers Gb4Cer (but can also bind Gb3Cer) [31]. Several amino acids in the C-terminus of the B subunit are critical for determining receptor preference. When the double mutation Q64E/K66Q is made to the Stx2e B subunit, it loses its ability to bind Gb4Cer, and has a receptor preference analogous to Stx2a [32]. The B subunit of Stx2f has Q64/K66 like Stx2e, and can bind both Gb3-LPS and Gb4-LPS, which are mimics of Gb3Cer and Gb4Cer, respectively [33].

Most Stx2 detection kits (both PCR and immunoassays) are optimized to Stx2a, and cross-react with closely related Stx2c and Stx2d. However, many do not recognize the divergent Stx2b, Stx2e, and Stx2f subtypes. Antibodies that recognize Stx2f have been reported, but few are commercially available and they are generally sold only as components of an assay kit, making them difficult to use as research tools and very expensive. Whether there is a reliable immunological method for detecting Stx2f is still a matter for debate. One of the primary means for detecting Stx1 and Stx2, the Premier EHEC kit from Meridian Biosciences, has been reported to detect Stx2f in two studies [30,34] but is insensitive to Stx2f in another [6]. A reverse passive latex agglutination assay (VTEC-RPLA) has repeatedly been shown to recognize Stx2f, but the sensitivity of this assay to Stx2f is unknown (Denka Seiken, Japan) [30]. In this study, we detail and characterize a group of novel monoclonal antibodies (mAbs) that react robustly and uniquely to Stx2f. With these antibodies, we have developed an immunoassay for simple detection of the Stx2f subtype.

## Materials and Methods

### Ethics Statement

All procedures with animals were carried out according to institutional guidelines for husbandry approved by the Animal Care and Use Committee of the U.S. Department of Agriculture, Western Regional Research Center (USDA ACUC Protocol 09-J-10). Mice were euthanized using rapid cervical dislocation to minimize suffering.

### *E. coli* strains and growth conditions

Strains expressing Stx2a (RM10638) and Stx2f (RM7007) as well as a control strain (K12) were grown as previously described [33]. Briefly, *E. coli* strains were inoculated into 10 mL of LB overnight at 37°C with agitation, then diluted 1/10 into 500 mL LB with 50 ng/mL mitomycin C (MMC) (Sigma-Aldrich, St. Louis, MO) and grown in a shaking incubator for 24 hours at 37°C. Cells were centrifuged for 15 minutes at 5000xG, the cell pellet was autoclaved, bleached, and discarded, and the media was sterile filtered. Stx2b (RM7005), 2c (RM10058), 2d (RM8013), 2e (RM7988), and 2g (10468)-expressing stains were also grown in this manner. Cells expressing the His-tagged Stx2f A subunit were also grown as described, as were Gb3-LPS- and Gb4-LPS-expressing strains [33]. FSIS EC465-97 is a wild-type *E. coli* O157:H7 strain transformed with pGFP which produces green fluorescent protein. It was provided by Todd J. Ward at the USDA-ARS, NCAUR, Peoria, IL 61604. All strains used in this study are listed in Table 1.

**Table 1:** *E. coli* strains used in this study.

Strain	Other names	Serotype	Biomolecule expressed	Origin	Reference
RM10638		O157:H7	Stx2a	Cow (2009)	[43]
RM7005	EH250	O188:H12	Stx2b	Clinical	[43]
RM10058		O157:H7	Stx2c	Bird (2009)	[43]
RM8013		ND^a^	Stx2d	Cow (2008)	[43]
RM7988		ND^a^	Stx2e	Water (2008)	This study
RM7007	T4/97	O128:H2	Stx2f	Feral pigeon	[43]
RM10468		ND^a^	Stx2g	Cow (2009)	[43]
RM5034	K12				[43]
CWG308 pJCP-Gb3			Gb3-LPS		[44]
CWG308 pJCP-*lgt*CDE			Gb4-LPS		[45]
CWG308					[44]
TOP10					Invitrogen
TOP10 pTrcHis2-Stx2fA			Stx2f A subunit +6xHis		[33]
FSIS EC465-97		O157:H7	*GFP-positive* Stx-negative	USDA, FSIS	[42]

^a^ Not determined.

### Purification of Stx2a and Stx2f

Purifications were conducted using cell-free supernatants of Stx2a (RM10638) and Stx2f-expressing (RM7007) *E. coli strains* and previously published protocols [33]. Recombinant His-tagged Stx2f A subunit was purified as previously described [33]. Partially purified (^≈^50% pure) Stx1 was purchased from Toxin Technologies (Sarasota, FL).

### Cell culture

Complete hybridoma media (cHM) used for the culturing of SP2/0 mouse myeloma cells and hybridoma cell lines consisted of Iscove’s modified Dulbecco’s Minimal medium (Sigma-Aldrich) containing NaHCO_3_ (36mM) and 1x Glutamax (Invitrogen, Carlsbad, CA), supplemented with 10% heat-inactivated fetal calf serum (FCS) (Invitrogen). Incomplete hybridoma media (iHM) is cHM without FCS. HAT (hypoxanthine, amniopterin, and thymidine) selection medium was prepared as 1x HAT supplement (Sigma-Aldrich) dissolved in cHM. Macrophage conditioned medium (MPCM) was prepared as previously described [35]. cHM was supplemented with 50% MPCM for the initial HAT selection and 10% MPCM for the hybridoma cloning steps. cHM with 1x HT (Hypoxanthine and thymidine, Sigma-Aldrich) was used during the first and second cloning steps. Cells were maintained at 37°C, 5% CO_2_.

### Immunization and polyclonal serum production

Mouse immunizations were conducted using His-tagged Stx2f A subunit as described previously [36]. Briefly, female Balb/cJ mice were injected intraperitoneally three times with 5 µg of Stx2f A-subunit in Sigma adjuvant system (Sigma-Aldrich) at two-week intervals, then bled (using the tail vein procedure) to collect polyclonal serum and confirm that the serum possesses antibodies that recognize Stx2f by direct ELISA using Stx2f purified from a bacterial strain as an antigen. The mouse with the highest anti-Stx2f serum titre was then boosted once with 1 µg Stx2f A subunit without adjuvant a week later. Three days later, the spleen was excised aseptically after euthanasia.

### Hybridoma development, cloning, and screening

Monoclonal antibodies (mAbs) were produced as described [36]. Briefly, cell fusions were achieved using SP2/0 myeloma cells, splenocytes extracted from the inoculated mouse spleen, and polyethylene glycol. Following fusion, the cells were diluted into ten 96-well plates and allowed to recover for 12 days in 50% MPCM/HAT/cHM medium. The hybridomas were then screened for antibodies recognizing Stx2f by ELISA and positive wells were transferred to 24-well plates in 10% MPCM/HT/cHM media to recover. Following recovery, the hybridomas were diluted to 500 cells/mL then serial diluted (2-fold) across a 96-well plate. This cloning step was repeated two additional times, with the final cloning being conducted in 10% MPCM/cHM. After clonal hybridoma lines were isolated, cells were grown in cHM media.

### Monoclonal antibody preparation

Around 400 mL of antibody-containing media (hybridoma cells grown in cHM for 2-3 days) was passed through a Protein G column (GE Healthcare). Antibody was eluted with 0.1 M glycine (pH 2.7), resulting in 4-6 mg of purified Stx2f antibody. Protein concentration was determined using the BCA Protein Assay Kit (Thermo Scientific, Rockford, IL). Biotinylation of antibodies was performed using the Lightning-Link Biotin Conjugation Kit (Innova Biosciences, Cambridge, UK). Antibody isotyping was conducted by ELISA using Stx2f and horseradish peroxidase (HRP) -conjugated isotype-specific antibodies (Southern Biotech, Birmingham, AL).

### Enzyme-linked immunosobent assays (ELISA)

**Figure 1:**
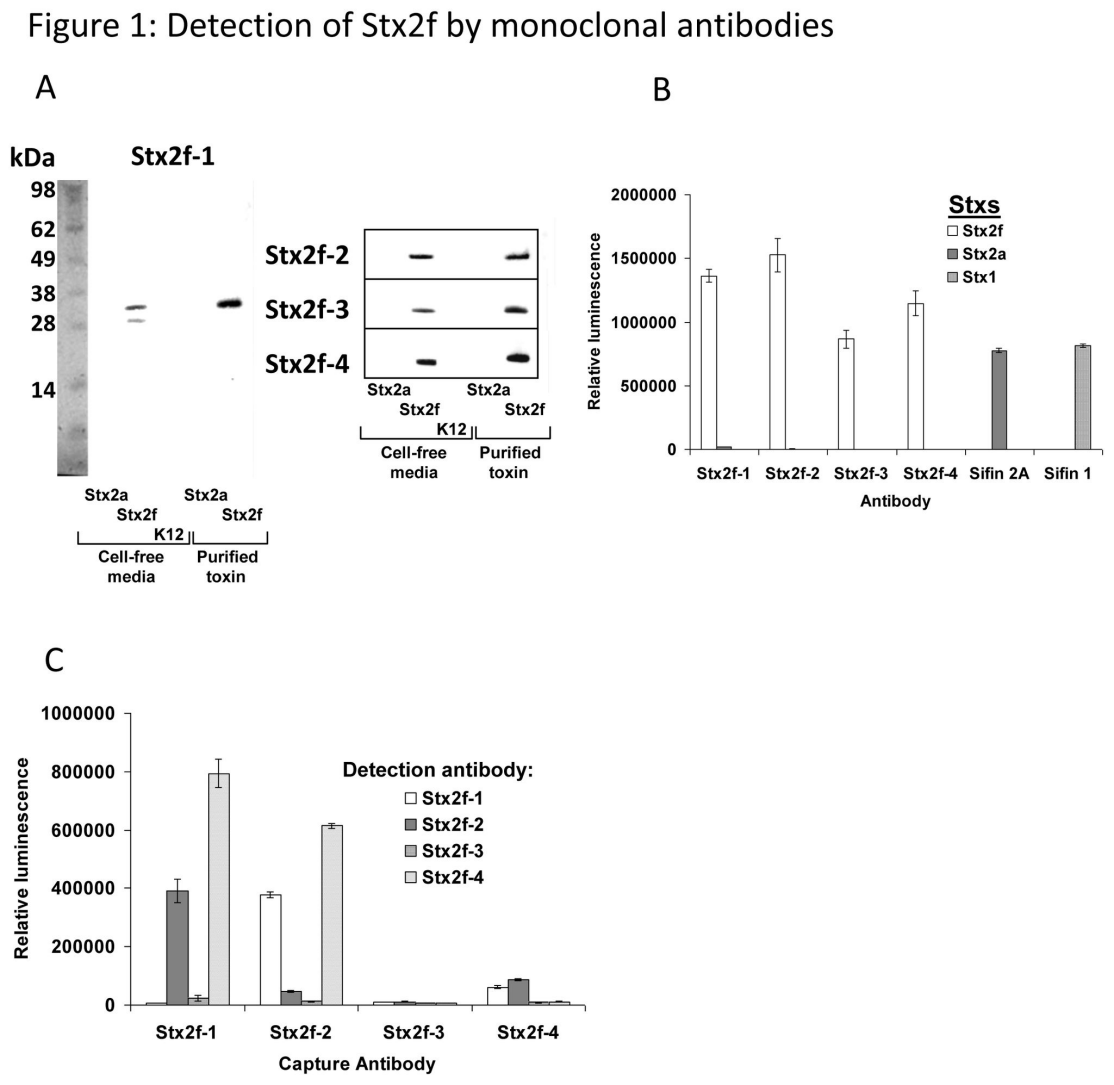
Detection of Stx2f by western blot analysis. A. Undiluted mitomycin C-induced (50 ng/mL) bacterial supernatants containing Stx2f, Stx2a, and K12 were loaded at 5 μL/lane. Purified Stx2f and Stx2a proteins were loaded at 5 ng/lane. Proteins were separated by SDS-PAGE. Membranes were probed with mAbs Stx2f-1, Stx2f-2, Stx2f-3, and Stx2f-4, respectively. Representative blots are shown (N=5). B. Direct ELISA for detection of Stx2f using Stx2f mAbs indicated. A concentration of 250 ng/mL purified Stx2f, Stx2a, and partially purified Stx1 toxin were used to coat the ELISA wells. The concentrations of mAbs and goat anti-mouse-HRP used were 1 µg/mL and 0.2 µg/mL, respectively. Sifin 2A (a mAb specific to Stx2) and Sifin 1 (a mAb specific to Stx1) were included as positive controls. The data shown represent the mean ± SD of three replicates from one representative experiment. Three individual experiments were performed. C. Stx2f sandwich ELISAs comparing different Stx2f antibody pairs. Coating antibodies and biotinylated detection antibodies were used at 1 µg/mL, streptavidin-HRP conjugate was used at 0.1 µg/mL, and the antigen (purified Stx2f) was used at 10 ng/mL. The data shown represent the mean ± SD of three replicates from one representative experiment. Three individual experiments were performed.

For hybridoma screening, Stx2f (50 ng/mL in Phosphate buffered saline [PBS]) was bound to the wells of a black NUNC Maxisorb 96-well plate overnight at 4°C. The plates were washed twice with PBS/0.05% Tween 20 (PBST) (using a BioTek ELx405 plate washer) and blocked with 200 μL/well 5% nonfat dry milk in PBST (blocking solution) for 1 hour at room temperature (RT). The plates were then washed twice with PBST, then 50 μL/well blocking solution was added to 50 μL/well hybridoma culture media. This was incubated for 1 hour at RT, followed by six washes with PBST. A 1/5,000 dilution of HRP-conjugated goat anti-mouse IgG antibody (GAM-HRP) antibody (Promega) in blocking solution was then dispensed into the plates, and incubated for 1 hour at RT. The plates were washed a further six times with PBST, then 100 μL/well Pico chemiluminescent substate (Thermo Scientific) was added, and 5 minutes later, luminescence was measured using a Victor II plate reader (PerkinElmer). Direct-well binding ELISAs (Figure 1B) were conducted in the same manner, except that 250 ng/mL Stx2f, Stx2a, and Stx1 was used to coat ELISA plates.

For sandwich ELISAs, purified capture antibody at 1 µg/mL in PBS was incubated in black Maxisorb 96-well plates overnight at 4°C. The plates were blocked and washed as with the hybridoma screening ELISA, except using 3% BSA in lieu of 5% nonfat dry milk. Plates were washed twice with PBST, then Stx2f (diluted in PBS to various concentrations) was then added at 100 μL/well, incubated for 1 hour at RT, and washed six times with PBST. Biotinylated antibody was diluted to 1 µg/mL, added to the plate at 100 μL/well, and incubated for 1 hour at RT, then the plates were washed six times with PBST. 1 mg/milliliter streptavidin-HRP conjugate (Invitrogen) was diluted to 1/10,000 in PBS, added at 100 μL/well, and incubated for 1 hour at RT. Following another six washes with PBST, the plates were developed and read like the hybridoma screening ELISAs. Limit of detection (LOD) was determined by extrapolating ng/mL of Stx2f from the background luminescence plus 3 standard deviations of the background. For chicken breast extract ELISAs, 0.25 g chicken breast was combined with 0.5 mL PBS and homogenized using a pestle in a microfuge tube. Debris was removed by centrifugation (12 kG, 5 min.), and the resulting suspension was sterile filtered (0.2 µm). This chicken breast extract was diluted 10-fold in PBS, then used to dilute Stx2f during the toxin binding step.

### Western blots

Western blots were conducted as previously described [33]. Samples were incubated at 72°C for 10 minutes in 1x NuPage SDS loading buffer before being run on a 4%–12% NuPAGE Novex Bis-Tris mini gel (Invitrogen). Then the proteins were transferred to a PVDF membrane (pore size, 0.45 µm; Amersham Hybond-P), blocked with 2% ECL Prime blocking agent (GE Healthcare) in PBST for 1 hour at RT, and washed thrice with PBST (3 minutes each). Antibodies were diluted 1/1000 in blocking solution and incubated with the blots for 1 hour at RT, then the blots were washed thrice again in PBST. GAM-HRP antibody (Promega) at a 1/10,000 dilution was incubated on the blot for 1 hour at RT, the blots were washed four more times with PBST, and developed using Lumigen TMA-6 (Lumigen) substrate. The blots were visualized with a 2 minute exposure using a FluorChem HD2 (Alpha Innotech).

### Gb3/4-LPS binding assays

Mouse mAb against Stx2a B-subunit (VT136/8-H4 from Sifin Institute, Berlin, Germany) or mAb Stx2f-1 (for Stx2f) at 1 µg/mL in PBS were bound to black Nunc Maxisorp plates and incubated overnight at 4°C. The plates were washed twice with PBST and blocked with 200 μL/well 3% BSA in PBST for 1 hour at room temperature (RT). During the blocking step, 125 ng/mL Stx2a and Stx2f toxin was incubated in microfuge tubes with varying amounts of Gb3-LPS or Gb4-LPS formalin-fixed cells diluted in PBS for 1 hour at RT. The toxin/cell complex was then spun down (12 k RPM for 2 minutes), and the liquid portion (containing unbound free toxin) in the microfuge tubes was then dispensed onto the blocked plates (100 μL/well) and incubated for 1 hour at RT. Plates were washed six times with PBST, then 1 µg/mL biotinylated detection antibody diluted in BSA blocking solution was added (biotinylated mAb Stx2f-4 was used for detection of Stx2f; biotinylated mAb VT135/6-B9 was used for detection of Stx2a) and allowed to incubate for 1 hour at RT. After another six washes, streptavidin-HRP conjugate was added at 1/10,000 in BSA blocking solution for 1 hour at RT. Following another six washes with PBST, the plates were developed and read in the same way as the hybridoma screening ELISAs above. There is an inverse relationship between the ELISA signal obtained and the amount of Stxs bound to Gb3-LPS and Gb4-LPS cells, since these cells remove the toxin from solution. The ELISA signal for Stx2a and Stx2f incubated without the presence of cells was initially set to 100% because all toxins were available to bind to the capture and detection antibodies for signal development. The ELISA signal for Stx2a and Stx2f incubated with Gb3 or Gb4 cells at an A_600_ of 0.2 was initially set to 0% because at this condition there is almost no toxin left to bind to the detection antibody; all the toxin bound to cells and was removed by centrifugation. Since we wanted to display binding of Stx to Gb3/4-LPS, we then flipped the values (100% signal became 0% binding, 0% signal became 100% binding, etc.). 50% binding was determined by calculation off a three point linear curve (points at A_600_ 0.067, 0.022, and 0.0074).

### Neutralization of Stx2f mediated cytotoxicity in Vero cells

Vero (African green monkey kidney) cells [18] were prepared as previously described [33]. Briefly, Vero cells were dispensed into 96-well cell culture plates at 10^5^ cells/mL overnight. The media used was Dubecco’s Modified Eagle’s Medium (DMEM) plus 1x Glutamax (Invitrogen) and 10% FBS (Invitrogen). Cells were treated at 4°C for 1 hour with 100 µl/well of Stx2f (5 ng/mL) or Stx2f pre-incubated with mAbs (100 µg/mL in Vero cell media) for one hour at RT. The media containing unbound toxin was then removed and replaced by fresh media, and cells were shifted to 37°C to grow for 24 hours. The cells were then lysed using 100 µl/well 1/5 dilution of CellTitre-Glo reagent (Promega), and luminescence was measured using a Victor II plate reader. The CellTiter-Glo Assay relies on the properties of a thermostable luciferase, which generates a stable luminescent signal in the presence of ATP and luciferin. The luminescent signal is proportional to the amount of ATP present, while the ATP is directly proportional to the number of metabolizing cells present in culture. The wells containing only 5 ng/mL toxin (without mAbs) were defined as 100% cytotoxicity or 0% neutralization and the negative control (no antibody or toxin) was set to 0% cytotoxicity (100% cell viability). Photos were taken using a Leica DM IL microscope at 200x magnification (Figure S1B). For Stx2 subtype treatments of Vero cells (Figure S2), strains expressing all seven subtypes of Stx2 were induced with 50 ng/mL MMC. The media was centrifuged to remove bacterial cells, then filter-sterilized (0.2 µm). Cell-free media (5 μL/well) containing Stxs was added to Vero cells, incubated for 1 hour at 4°C, and replaced with fresh media. Photos were taken using a Leica DM IL microscope at 200x magnification (Figure S2).

### Antibody affinity measurement

Antibody affinity to Stx2f was measured using an Octet QK system (Forte-bio, Menlo Park, CA) as described previously [36]. The biotinylated antibodies were coupled to streptavidin biosensors at 10 µg/mL in PBS. Probes coupled to antibody were incubated with Stx2f at four different concentrations (142, 71, 36, and 18 nM), then allowed to dissociate in PBS. Binding kinetics were calculated using the Octet QK software (Data Acquisition 7.0).

### Colony immunoblots

Strains RM7007 (Stx2f) and FSIS EC465-97 (GFP-labeled O157:H7, Stx-negative) were grown in LB broth for 12 hours at 37°C with agitation. RM10638 (Stx2a) was additionally grown for Figure S3. Following this, the A_600_ of each of these cultures was set to 2 and 100 µL of each culture was combined (for a total volume of 200 µL [or 300 µL for Figure S3]). The mixture was diluted 10^6^ times in LB broth and 100 µL of this dilution was plated on LB agar plates supplemented with 50 ng/mL MMC, using sterile glass beads for distribution. The LB agar plates were incubated for 12 hours at 37°C. A rectangular cut of PVDF membrane was then wetted in methanol and incubated in water for 5 minutes. After blotting the membrane dry, it was placed upon the LB plate and incubated at 4°C for 2 hours. It was then incubated in a boiling hot 2% SDS solution for 5 minutes, and this step was repeated to kill all residual bacteria. The membrane was then rinsed three times in PBS, for 5 minutes each time with agitation to remove cell debris. The membrane was then blocked in 2% ECL Prime blocking agent/PBST for 1 hour at RT. Afterwards, the membrane was incubated with a solution of 1 µg/mL mAb Stx2f-4 in blocking solution for 1 hour at RT. Following this, the membrane was washed thrice (3 minutes each) with PBST then incubated with a 1/10,000 dilution of GAM-HRP (Promega) in blocking solution for 1 hour at RT. After 4 washes with PBST (5 minutes each), the blots were developed with Lumigen TMA-6 (Lumigen) substrate. Colony blots were visualized with a 2 minute exposure using a FluorChem HD2 (Alpha Innotech). Photos for plates were taken using an iPhone 4S and GFP-labeled control cells were illuminated on a UV box (U: Genius, Syngene, Cambridge, UK). The colony immunoblot was false-colored (red) in Photoshop (Adobe) to enhance contrast for an overlay picture. For plates supplemented with chicken breast extract (see the Enzyme-linked immunosobent assays section), 50 μL/plate extract was dispensed on LB plates containing 50 ng/mL MMC and allowed to absorb before plating 50 µL of the bacterial mixture (diluted 5 x 10^5^ in LB broth).

### Stx2a and Stx2f PCR

Diagnostic colony PCR (Figure S3B) was performed to confirm the specificity of the colony immunoblot assay for Figure S3A. Colonies were tapped with a pipet tip before performing the colony immunoblot, and the bacteria was suspended in 100 µL sterile water. PCR was performed using previously described primers and protocols [27], with a few modifications. The Stx2a PCR used the primers stx2a-F2 and a 1:1 combination of stx2a-R2 and stx2a-R3, for an amplicon of 347 or 349 base pairs. The Stx2f PCR used the primers stx2f-F1 and stx2f-R1, for an amplicon of 324 base pairs. A mixture of 20 µM primers (1.25 µL per 25 µL reaction), 2x GoTaq master mix (Promega) (12.5 µL per 25 µL reaction), bacterial suspension (1 µL of the suspension per 25 µL reaction), and water (up to 25 µL) was cycled 35 times, with an annealing temperature of 64°C.

## Results

### Producing Stx2f mAbs

To generate high-affinity mAbs against Stx2f, we immunized mice with purified recombinant His-tagged Stx2f A subunit (a listing of all bacterial strains used is included in Table 1) [33] and fused the resulting splenocyes to SP2/0 myeloma cells. Splenocyte/myeloma hybridoma fusions, plated into 96-well culture plates (960 wells total), were screened using purified Stx2f [33]. Thirty-seven wells were chosen for further analysis. After repeated expansion and isolation of cells by limiting dilution, four hybridoma cell lines were selected. The antibodies purified from these hybridoma cell lines are designated mAbs Stx2f-1, Stx2f-2, Stx2f-3, and Stx2f-4, and all possessed IgG2 except mAb Stx2f-2 which has an IgG1 isotype (Table 2). All these antibodies bound specifically to the Stx2f A subunit (^≈^32kD) on a western blot and had no discernable affinity to the B subunit (^≈^5kD) (Figure 1A). All four antibodies bound strongly to purified Stx2f but not to purified Stx2a or partially purified Stx1 in a direct ELISA (Figure 1B). Mouse mAb VT135/6-B9 (for Stx2a) and VT109/4-E9b (mouse mAb against Stx1 B-subunit from Sifin) were included as controls to confirm the presence of these toxins. Dissociation constants for mAbs Stx2f-1 and Stx2f-2 (0.52 and 0.53 x 10^-9^ M, respectively) were considerably lower than that of Stx2f-4 (8.4 x 10^-9^ M), while no dissociation constant could be calculated for Stx2f-3 despite repeated attempts (Table 2).

**Table 2:** Properties of Stx2f monoclonal antibodies.

Antibody	Isotype	K_D_ (x 10^-9^ M)
Stx2f-1	IgG2, kappa	0.516 ± 0.14
Stx2f-2	IgG1, kappa	0.533 ± 0.39
Stx2f-3	IgG2, kappa	nd*
Stx2f-4	IgG2, kappa	8.35 ± 1.1

* Not detectable.

### Developing a sensitive and specific sandwich ELISA for Stx2f

**Figure 2:**
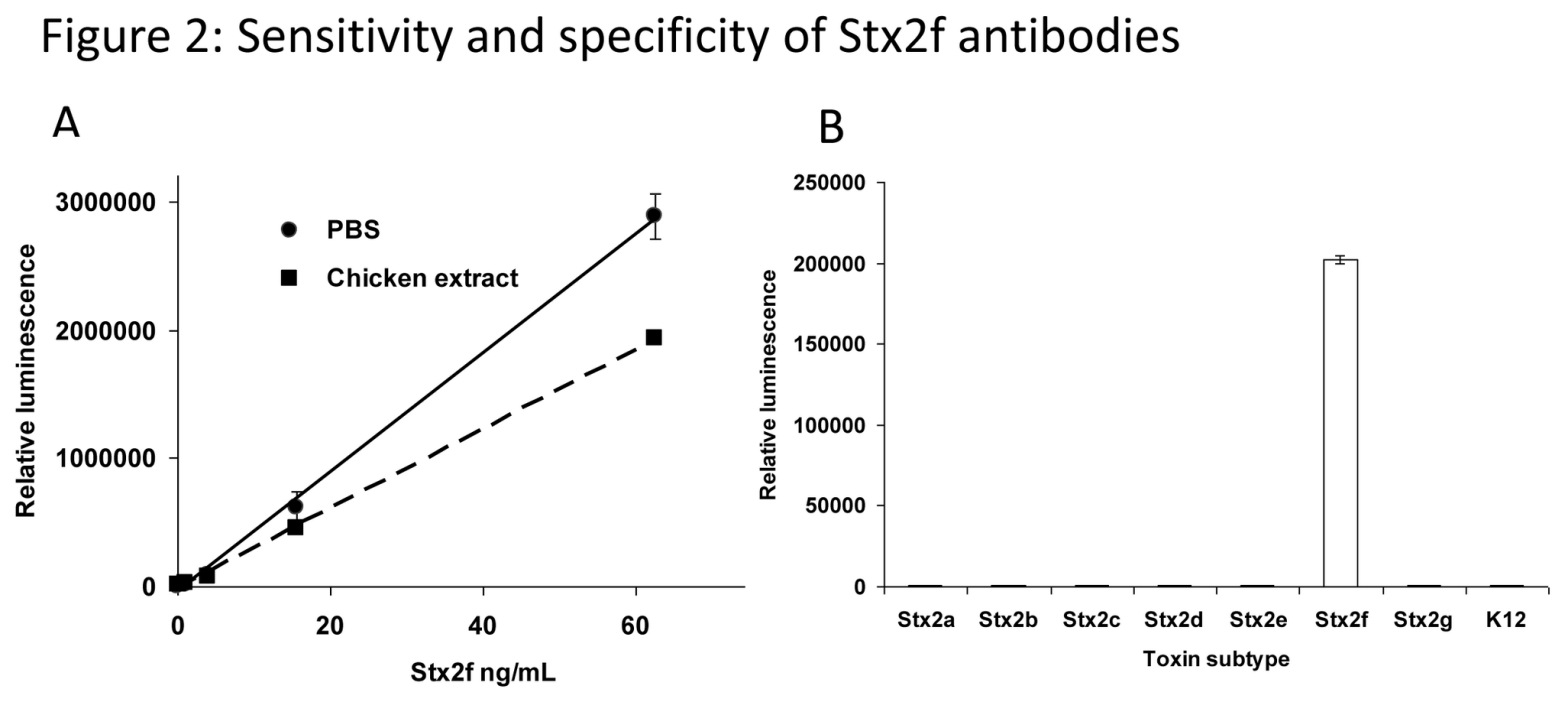
Sensitivity and specificity of Stx2f mAbs. A. Detection of Stx2f in PBS (●) or chicken extract (■) by ELISA using the mAb Stx2f-1 as capture and mAb Stx2f-4 as a detector. Purified Stx2f ranging from 0-60 µg/mL were used for this assay. The data shown represent the mean ± SD of three replicates from one representative experiment (this experiment was performed four times with similar results). B. The mAb Stx2f-1/4 sandwich ELISA reacts exclusively with Stx2f cell culture supernatant. Mitomycin C-induced cell-free bacterial supernatants (at a 2-fold dilution) for all seven subypes of Stx2 were prepared and analyzed by ELISA. The data shown represent the mean ± SD of three replicates from one representative experiment. Three individual experiments were performed.

In order to establish a sensitive immunoassay, all possible capture/detector combinations of mAb pairs were evaluated in a sandwich ELISA format using a biotinylated antibody as a detector. The following capture/detector antibody pairs are highly effective at detecting Stx2f purified toxin: mAb Stx2f-1/2, Stx2f-1/4, Stx2f-2/1 and Stx2f-2/4 (Figure 1C). mAbs Stx2f-1 and Stx2f-2 were very effective as capture antibodies, Stx2f-4 was the best detector, and Stx2f-3 was not compatible with any of the other Stx2f antibodies, either as a capture or detection antibody. The most sensitive antibody pair employed mAb Stx2f-1 as a capture antibody and Stx2f-4 as a detector. This pair detected purified toxin down to 0.123 ng/mL (Figure 2A). In addition, over the range of toxin tested (0-60 ng/mL), the assay was linear, with an R^2^ value of 0.9979. The specificity of the mAb Stx2f-1/Stx2f-4 pair was evaluated using filtered cell culture media containing different Stx2 subtypes, induced with MMC (purified toxin is not available for many of these subtypes). The MMC-induced media from all seven subtypes of Stx2 was toxic to Vero cells, confirming the presence of toxin (Figure S2). The mAb Stx2f-1/4 ELISA did not recognize any other Stx2 subtype tested, suggesting that this combination of antibodies is specific to Stx2f (Figure 2B).

Poultry is an emerging source of *E. coli* contamination [37,38]. Since Stx2f-producing *E. coli* has been isolated from an avian vector (pigeon), it is reasonable to assume that Stx2f-producing bacteria may soon be associated with poultry meat. We therefore examined the effect of chicken extract on the Stx2f sandwich ELISA assay (mAb Stx2f-1/4). Stx2f was spiked in chicken breast extract (1/10 dilution in PBS). Although the overall signal of the sandwich ELISA was reduced somewhat, this had little detrimental impact upon the limit of detection for Stx2f, which rose to 0.210 ng/mL (Figure 2A).

### Examining the preference of Stx2f to receptor using Stx2f mAbs

**Figure 3:**
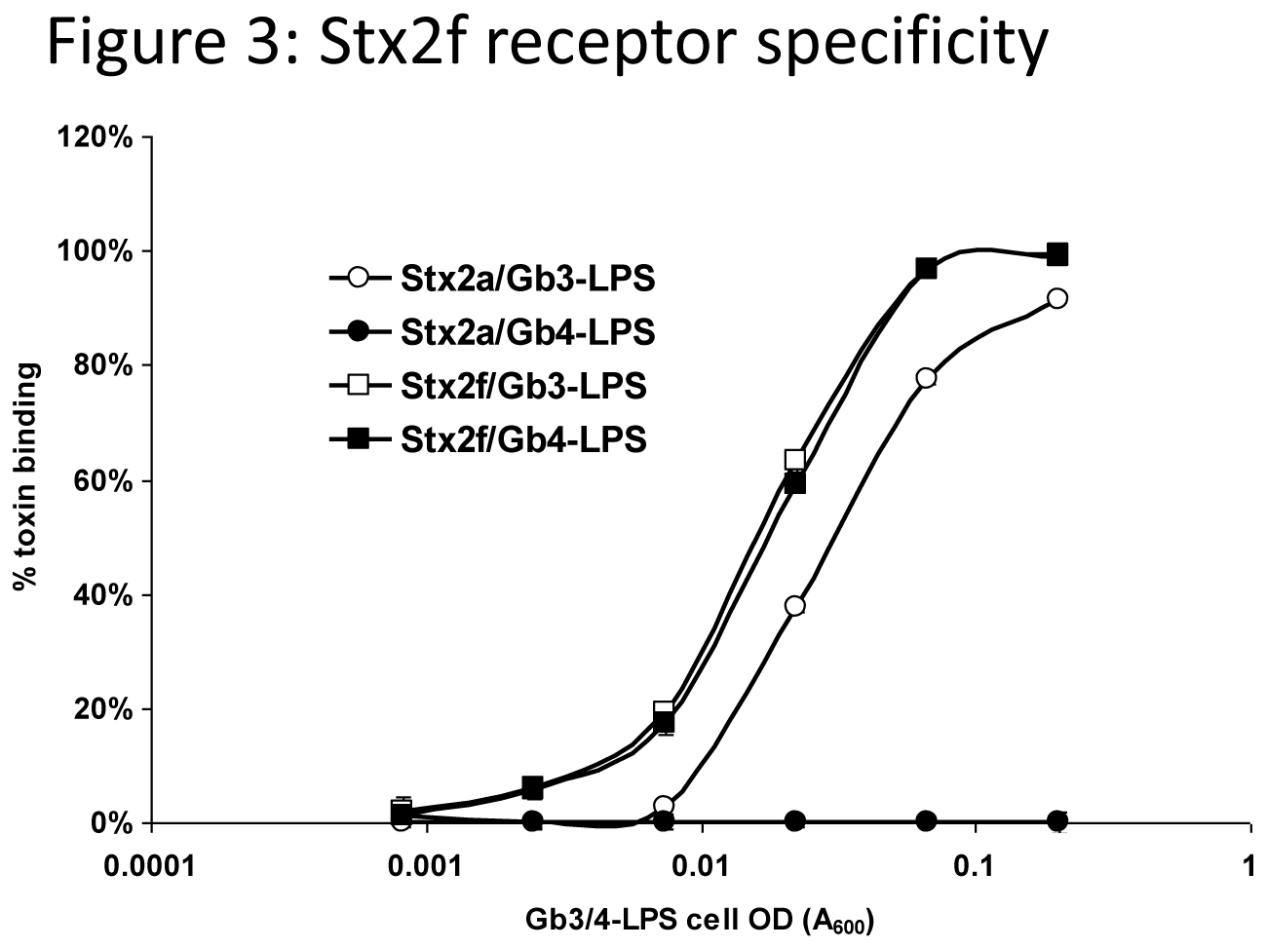
Binding of Stx2f to Gb3-LPS and Gb4-LPS receptors. Various amounts of Gb3-LPS- or Gb4-LPS-expressing cells were mixed with a fixed amount of purified Stx2f or Stx2a (250 pg/mL) in a microtube. Unbound toxins were recovered after centrifugation and quantified by ELISA using a mAb pair (Stx2f-1/4 for Stx2f or Sifin A/B for Stx2a). Stx2f binds to Gb3-LPS and Gb4-LPS cells with equal affinity, while Stx2a binds only Gb3-LPS cells. The average of three replicates of a representative experiment is shown (this experiment was conducted three times).

Our previous results suggest that Stx2f is able to bind both Gb3- and Gb4-LPS receptors. Using the Stx2f-1/4 antibody pair, it was possible for us to perform sandwich ELISAs to confirm the receptor preference of Stx2f. Gb3-LPS or Gb4-LPS-expressing *E. coli* cells were pre-incubated with Stx2a or Stx2f toxin, the cells were then removed, and the remaining toxin was quantified by ELISA using the corresponding coating/detection antibody combination (mAb Stx2f-1/4 for Stx2f and mAb VT136/8-H4/VT135/6-B9 for Stx2a). With 50% of toxin bound at an A_600_ of 0.017 for Gb3-LPS and 0.018 A_600_ for Gb4-LPS, Stx2f bound strongly to both Gb3-LPS and Gb4-LPS receptors. Stx2a only bound to Gb3-LPS in this assay, with 50% bound at an A_600_ of 0.03 (Figure 3). Control cells (CWG308, Table 1) did not bind either Stx2a or Stx2f (data not shown [33]).

### *In vitro* toxin neutralization

**Figure 4:**
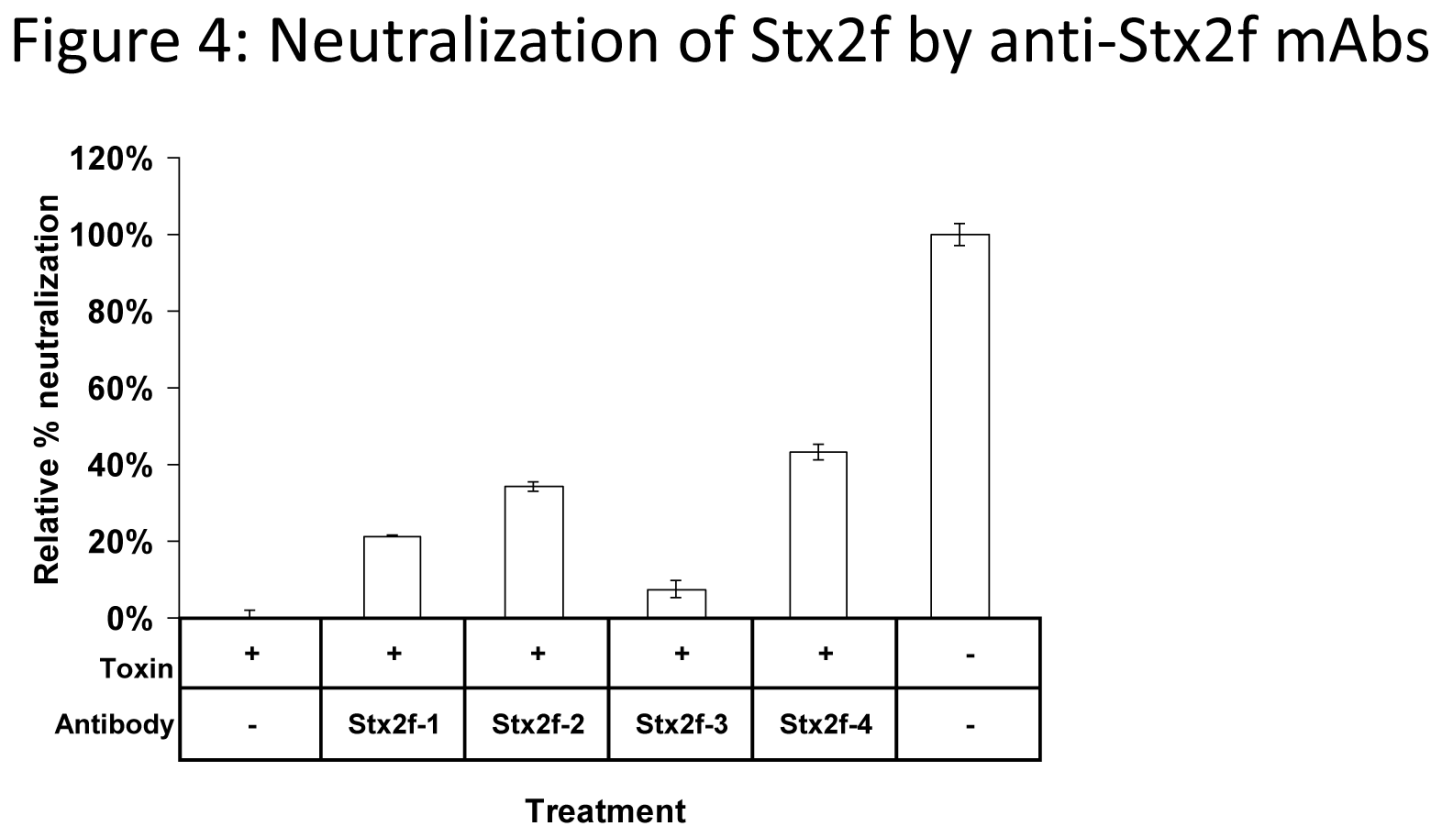
Neutralization of Stx2f by anti-Stx2f mAbs. Stx2f (5 ng/mL) was pre-incubated with antibody (100 µg/mL) for 1 hour at RT. This mixture was then incubated with Vero cells for 1 hour at 4°C. The media was removed and new media was added. Cell viability was measured using the CellTitre-Glo reagent. Data shown represent the mean ± SD of three replicates from one representative experiment. This experiment was conducted three times with similar results.

Antibodies against Stx2 B subunits tend to possess stronger neutralizing potential in cell-based assays than those against the A subunit [36], presumably by disrupting the binding of the toxin to Gb3/4 binding sites. However, antibodies against the A subunit that can reduce the *N*-glycosidase activity of Stx2 and provide some toxin neutralizing activity have been reported [39]. Therefore, we investigated whether our panel of Stx2f antibodies, administered at a 100 µg/mL concentration, can protect Vero cells from Stx2f toxicity. Though none of our antibodies conferred full protection from Stx2f, three of the four antibodies partially mitigated toxicity, with the best being mAb Stx2f-4 at 43% neutralization (Figure 4). These antibodies were about two-thirds as effective at neutralizing toxin at a lower concentration (10 µg/mL) (data not shown). In some circumstances, different partially neutralizing antibodies can synergize and strongly neutralize when combined [40]. While the combination of mAbs Stx2f-1 and Stx2-4 (the best sandwich ELISA combination) did not fully neutralize Stx2f, it did protect better than either of these antibodies separately, at 62% neutralization (Figure S1A, S1B).

### Identification of Stx2f-producing *E. coli* present in chicken extracts by colony immunoblot assay

**Figure 5:**
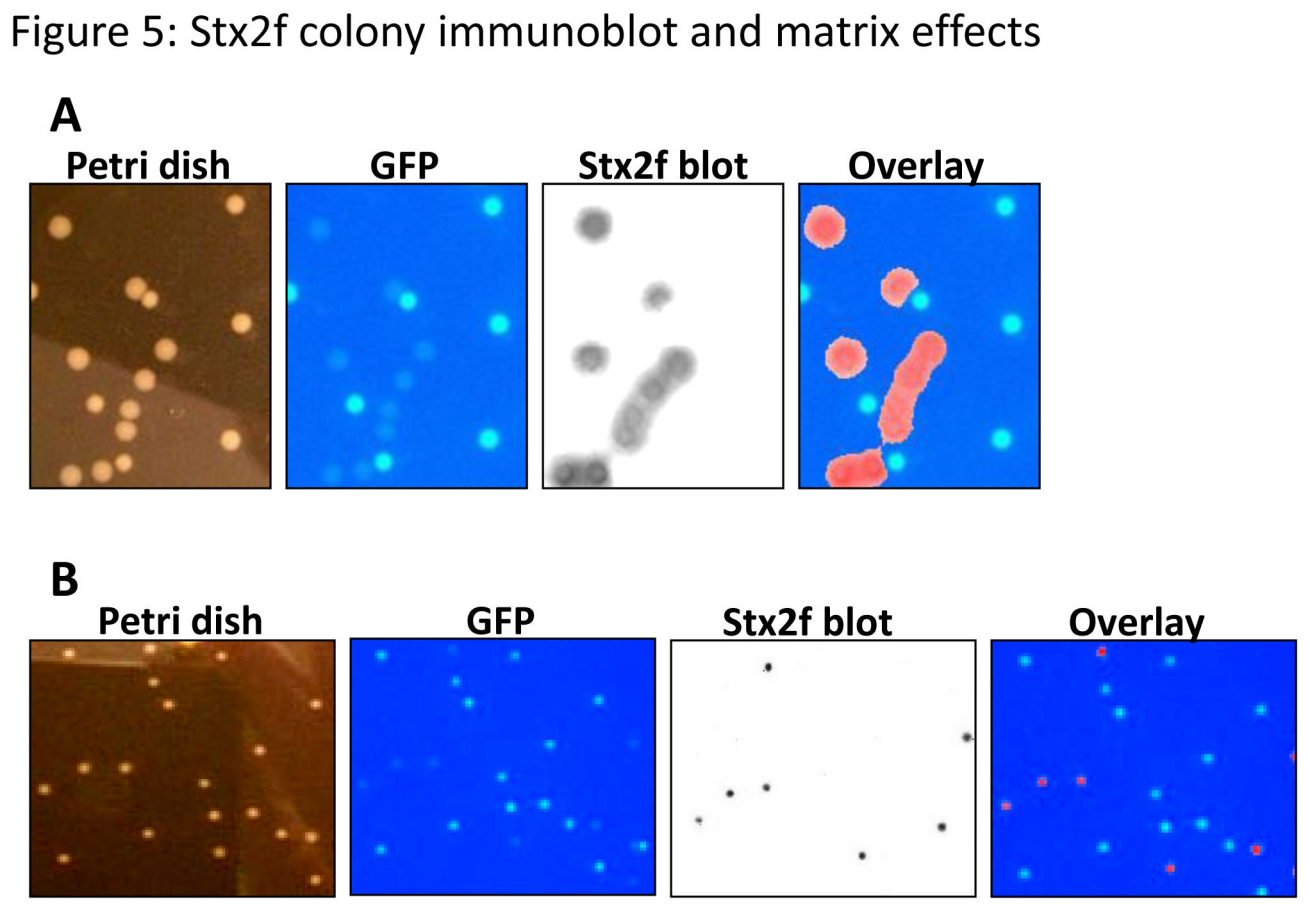
Stx2f colony immunoblot and matrix effects. A. A Stx2f colony immunoblot using mAb Stx2f-4 was conducted upon a mixture of Stx2f-expressing cells and GFP-labeled control cells. The cells were diluted 10^6^ and plated on LB +50 ng/mL mitomycin C. The same petri dish portion is displayed for all four panels (Petri dish, GFP, Stx2f blot, and Overlay). B. A Stx2f colony immunoblot is shown using the same mixture of cells as in 5A, plated on an LB plate containing mitomycin C and supplemented with 50 µL of chicken breast extract. The same petri dish portion is displayed for these four panels.

Detecting STEC in environmental or clinical samples is often a lengthy process, involving selection and isolation, usually followed by PCR or immunological confirmation of a pure culture of the organism. Since there is no accepted immunological assay for Stx2f and PCR does not reveal expression of the toxin, we sought to provide a plate-based method of detecting Stx2f-expressing *E. coli* using colony immunoblot assays. Growing STEC on agar plates supplemented with mitomycin C (MMC) is a way to maximize sensitivity in this type of assay [41]. We used a GFP-tagged O157:H7 marker strain (FSIS EC465-97 [42]), which has a genetic background analogous to STEC strains except it does not contain any functional *stx* genes, as a control and mixed it with the Stx2f-producing strain. Under our experimental conditions, all negative control colonies (fluorescent green) were not detected by mAb Stx2f-4 in the colony blot (Figure 5A-GFP), whereas all Stx2f-producing colonies which did not fluoresce were positive for Stx2f (Figure 5A-Stx2f blot and Overlay). To verify that the Stx2f-immunoblot assay does not cross-react with Stx2a-expressing colonies, we mixed the Stx2f-strain with the Stx2a- and GFP-strains and plated the mixture of these three strains on MMC plates. We then performed the Stx2f colony immunoblot, along with a colony PCR to detect the presence of the *stx2f* and *stx2a* genes. As we expected, every colony that was positive by Stx2f immunoblot was also positive for *stx2f* -PCR. Additionally, no Stx2a or GFP-O157:H7 colonies were detected by Stx2f immunoblot, meaning that this assay is specific to Stx2f colonies (Figure S3A, S3B).

Since poultry is a likely source of future contamination by Stx2f-expressing *E. coli*, sterile homogenized chicken breast extract was added to a subset of immunoblot plates to test for matrix effects. Surprisingly, both *E. coli* strains tested (Stx2f-producing *E. coli* and GFP-O157:H7) had smaller colonies, suggesting that the chicken breast was inhibiting their growth on plates (Figure S4), although this extract didn’t inhibit their growth in liquid media (data not shown). The Stx2f-producing bacteria could unambiguously be identified by colony immunoblot using our mAb Stx2f-4, (Figure 5B), however, suggesting that this colony immunoblot assay could be applied to poultry samples.

## Discussion

Since STEC are among the most dangerous foodborne pathogens, their detection and study is critical to maintaining a safe food supply and preventing deadly outbreaks. Stx2 is a particularly important virulence factor in EHEC and EHAEC, and is thought to be a causative agent in the development of HUS. The seven Stx2 subtypes, unlike those of Stx1, are very diverse, both at the amino acid and biochemical levels. Certain subtypes, like Stx2e and Stx2f, are not commonly associated with serious human disease, but this may be due to their current serotype distribution. Only a few immunoassays claim to detect Stx2f and the sensitivity and specificity of these assays is unclear. Here, we detail new mAbs for detecting the Stx2f subtype. The four antibodies developed in this study are specific to Stx2f and do not cross-react with Stx2a or Stx1. A sensitive new immunoassay for Stx2f was developed using a compatible pair of these Stx2f mAbs (Stx2f-1/Stx2f-4) in a sandwich ELISA format. The limit of detection for this new Stx2f immunoassay is 0.123 ng/mL and the specificity of this ELISA is to Stx2f uniquely: it does not cross-react with any of the other Stx2 subtypes. Using this ELISA, Stx2f was confirmed to bind to both Gb3-LPS and Gb4-LPS with similar affinities to each. Three of the four antibodies partially neutralize purified Stx2f toxin in a Vero cell viability assay, and the combination of Stx2f-1 and Stx2f-4 neutralize Stx2f even more effectively. Additionally, one of these new Stx2f antibodies (Stx2f-4) was validated for the detection of Stx2f-expressing colonies in a colony immunoblot assay, a technique that may prove useful in characterizing new Stx2f-expressing STEC isolates.

Although Stx2f-expressing STEC is not commonly detected in human disease, if and when the phage carrying the *stx2f* gene establishes itself in an *E. coli* strain that is capable of causing human disease, it could become a serious human pathogen. This new sandwich ELISA will be extremely valuable for future diagnosis and source tracking of contaminations. Some additional studies are needed to fully evaluate this assay and prepare it for use in food safety. A full evaluation of the effect of various common matrices (beef, milk, etc.) upon the sensitivity and reliability of this assay is currently underway. However, we predict that chicken or turkey meat is the most likely immediate source of contaminations, as Stx2f-producing strains were first isolated from an avian species (pigeon). Thus, chicken breast was used as a matrix for the Stx2f colony immunoblot and mAb Stx2f-1/4 sandwich ELISA, and both assays appear to be compatible with this matrix.

Ideally, ELISA assays for determining antibody sensitivity and specificity should be conducted using pure antigen, but bacterial supernatant containing Stx2 subtypes is frequently used as a toxin source. This is mainly due to the lack of pure toxin for some of the Stx2 subtypes. There are several drawbacks of using crude toxins in such studies. Most importantly, different STEC isolates and strains could exhibit different expression levels of Stx subtypes, which could easily explain contradictory results in previous studies [6,34]. Additionally, Stx-expressing isolates are very diverse, and some may possess molecules that interfere with an assay, generating false-positive or false-negative results. Western blotting is a highly informative assay that should be included in assay evaluations if pure antigen is unavailable.

The Stx2f colony immunoblot could greatly improve detection and isolation of any Stx2f-expressing bacteria, such as STEC, *Enterobacter cloacae*, which may express Stx as well [15], and *Escherichia* *albertii* , which may be a reservoir for the phage carrying Stx2f [14]. Strategies such as this and colony DNA hybridization are helpful for pinpointing specific STEC colonies in complex environmental or clinical samples and recovering it. An advantage of Stx colony immunoblotting over colony DNA hybridization is that the colony immunoblot can determine whether an STEC strain is actually expressing Stx, rather than just carrying the gene. Additionally, the signal of a colony immunoblot can be amplified using the appropriate culture conditions, in this case, supplementation with 50 ng/mL MMC [41]. Our Stx2f immunoblot detected only Stx2f-expressing colonies, not Stx2a-expressing colonies or toxin-free O157:H7 control strains (Figure 5A, Figure S3), even in the presence of chicken extract (Figure 5B). This suggests that the mAb Stx2f-4 colony immunoblot may be uniquely specific to Stx2f, and could be used to isolate Stx2f-producing bacteria regardless of the species, matrix, or presence of other Stx. Ultimately, we envision an all-encompassing colony immunoblot, ELISA, and/or lateral flow assay where all Stx subtypes can be detected separately from each other, in a wide variety of matrices. Although assays with wide ranges of specificity are very useful for monitoring the safety of food sources, an analogous subtype-specific kit would be even more valuable for tracking the migration prevalence of Stx subtypes and Stx-encoding phages.

## Supporting Information

Figure S1 A. Neutralization of Stx2f in a Vero cell assay with different combinations of mAbs against Stx2f. All neutralizations were conducted using 5 ng/mL purified Stx2f (except for the “No toxin” [PBS] control) and 100 µg/mL total concentration of mAbs. B. Microscope photographs are displayed for these assay wells with the indicated treatments.
(TIF)

Figure S2 **Stx2 subtype cytotoxicity.** Vero cells (seeded at 10^5^ cells/well and grown for 12 hours at 37°C) were treated with 5 μL/well bacterial cell-free supernatant (induced by 50 ng/mL MMC) containing the indicated Stx2 subtype for 16 hours at 37°C. All seven subtypes are expressed and are toxic to Vero cells.
(TIF)

Figure S3 A. Stx2f colony immunoblot with Stx2f- and Stx2a-expressing strains, as well as GFP-labeled control cells. The same plate portion is displayed for all four panels. B. Confirmation of the presence of the *stx2a* and *stx2f* genes by colony PCR. The Stx2a-specific PCR band is ~347 base pairs; the Stx2f-specific band is 424 base pairs. All colonies that are neither green (GFP) nor red (Stx2f-producing) are Stx2a-producing, confirmed by colony PCR (colony no. 1, 4, 7, 9, 12, and 14).
(TIF)

Figure S4 **The effect of chicken extract on colony size.** Adding 50 µL of chicken breast extract slows the growth of the Stx2f-expressing strain and the FSIS EC465-97 fluorescent control strain. The colonies on these plates are derived from the same two strain mixture as in Figure 5B.
(TIF)
