# Supplementary figures and images for: Development of Monoclonal Antibodies and Immunoassays for Sensitive and Specific Detection of Shiga Toxin Stx2f
Source: PLoS One. 2013 Sep 17;8(9):e76563. doi: 10.1371/journal.pone.0076563 (PMC3775747; doi:10.1371/journal.pone.0076563)

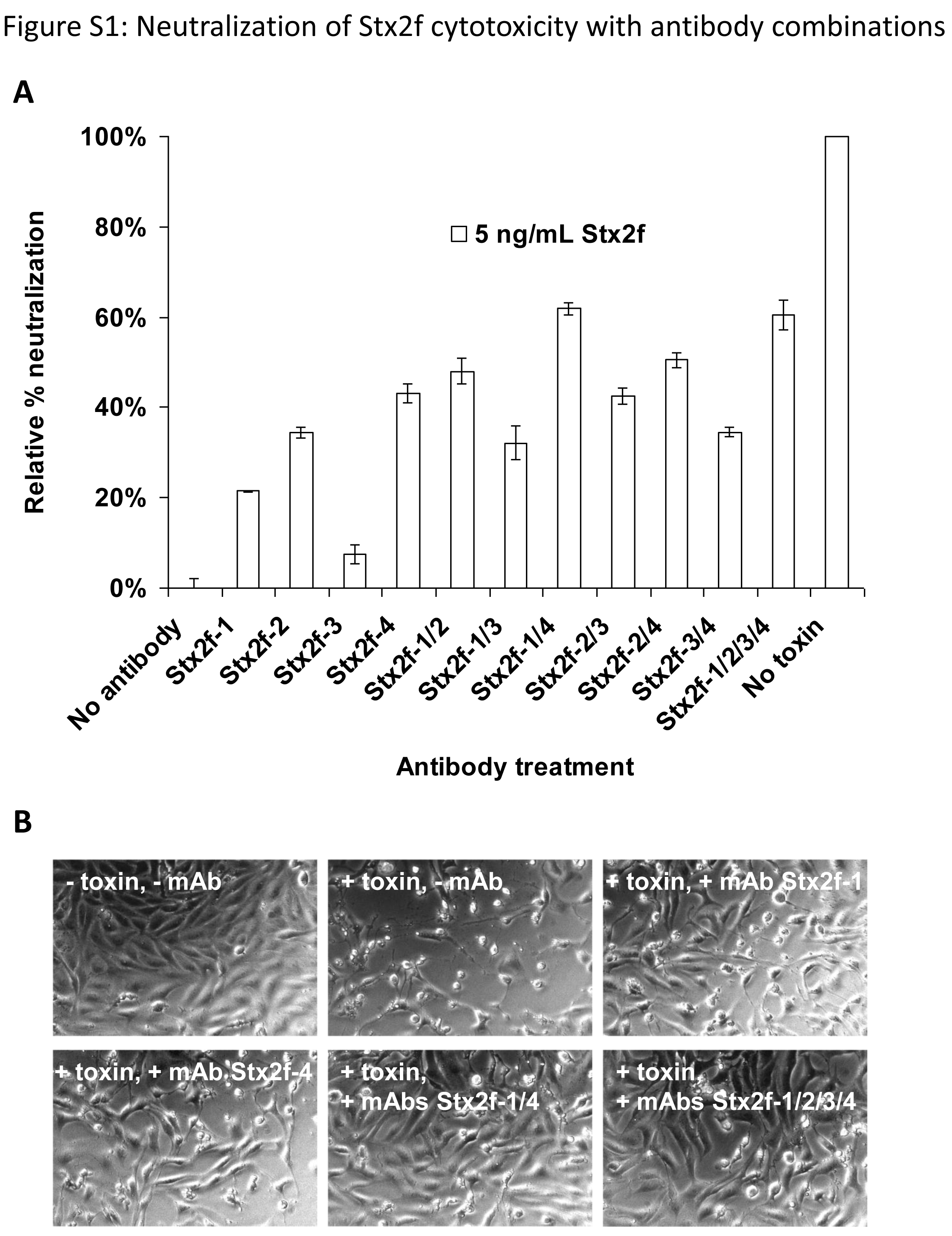

Supplement: Figure S1 — A. Neutralization of Stx2f in a Vero cell assay with different combinations of mAbs against Stx2f. All neutralizations were conducted using 5 ng/mL purified Stx2f (except for the “No toxin” [PBS] control) and 100 µg/mL total concentration of mAbs. B. Microscope photographs are displayed for these assay wells with the indicated treatments. (TIF) [file pone.0076563.s001.tif]

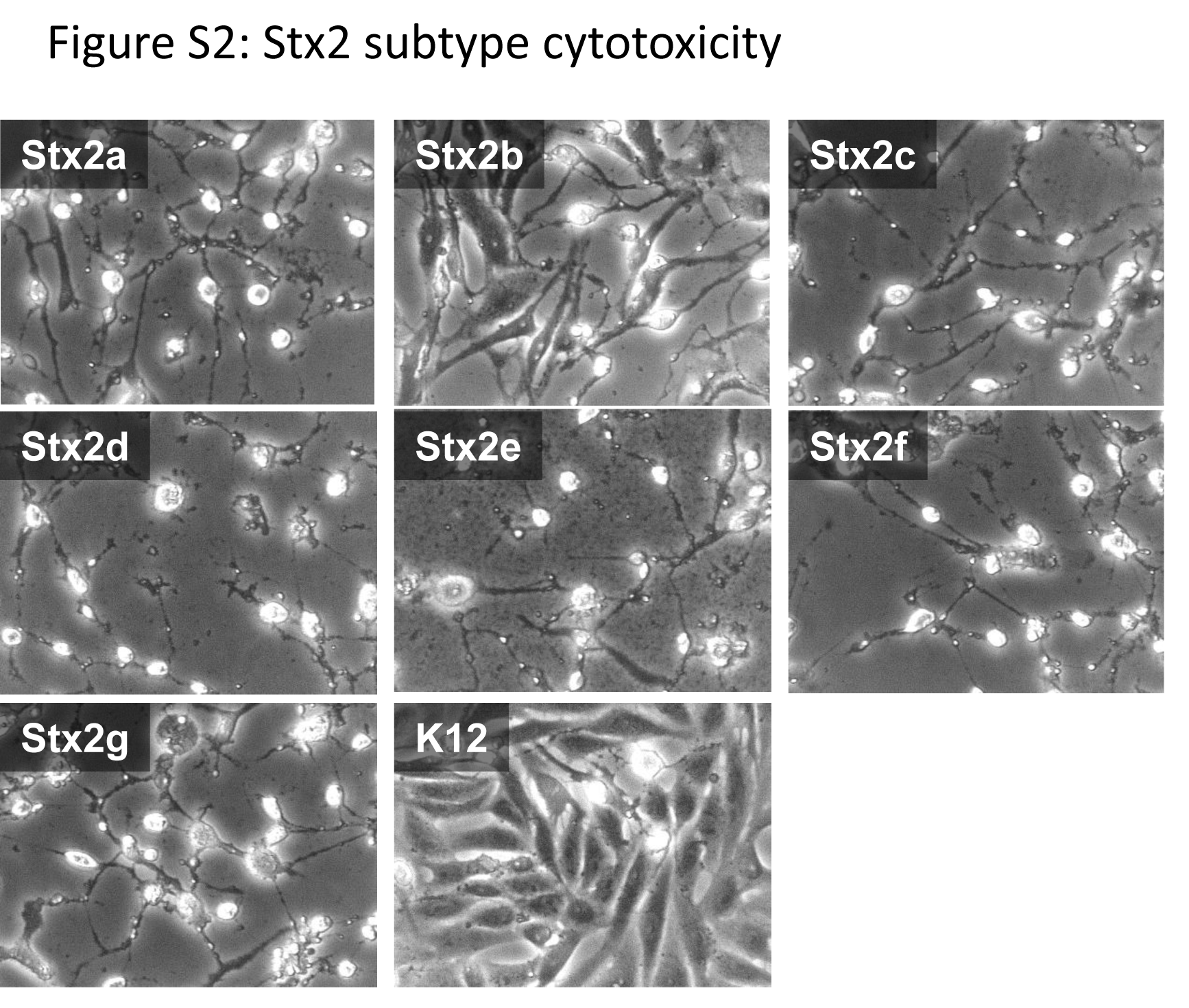

Supplement: Figure S2 — Stx2 subtype cytotoxicity. Vero cells (seeded at 105 cells/well and grown for 12 hours at 37°C) were treated with 5 μL/well bacterial cell-free supernatant (induced by 50 ng/mL MMC) containing the indicated Stx2 subtype for 16 hours at 37°C. All seven subtypes are expressed and are toxic to Vero cells. (TIF) [file pone.0076563.s002.tif]

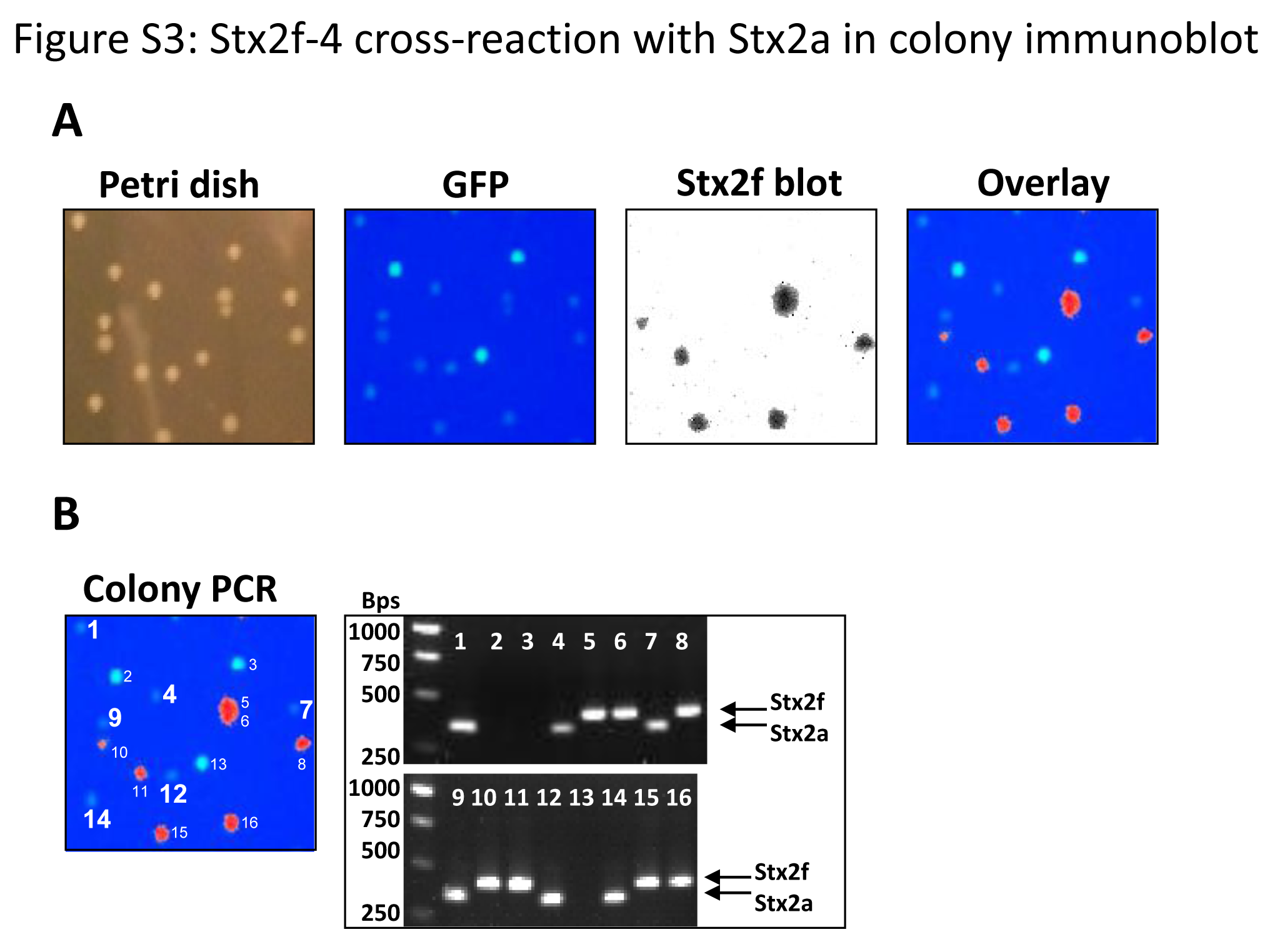

Supplement: Figure S3 — A. Stx2f colony immunoblot with Stx2f- and Stx2a-expressing strains, as well as GFP-labeled control cells. The same plate portion is displayed for all four panels. B. Confirmation of the presence of the stx2a and stx2f genes by colony PCR. The Stx2a-specific PCR band is ~347 base pairs; the Stx2f-specific band is 424 base pairs. All colonies that are neither green (GFP) nor red (Stx2f-producing) are Stx2a-producing, confirmed by colony PCR (colony no. 1, 4, 7, 9, 12, and 14). (TIF) [file pone.0076563.s003.tif]

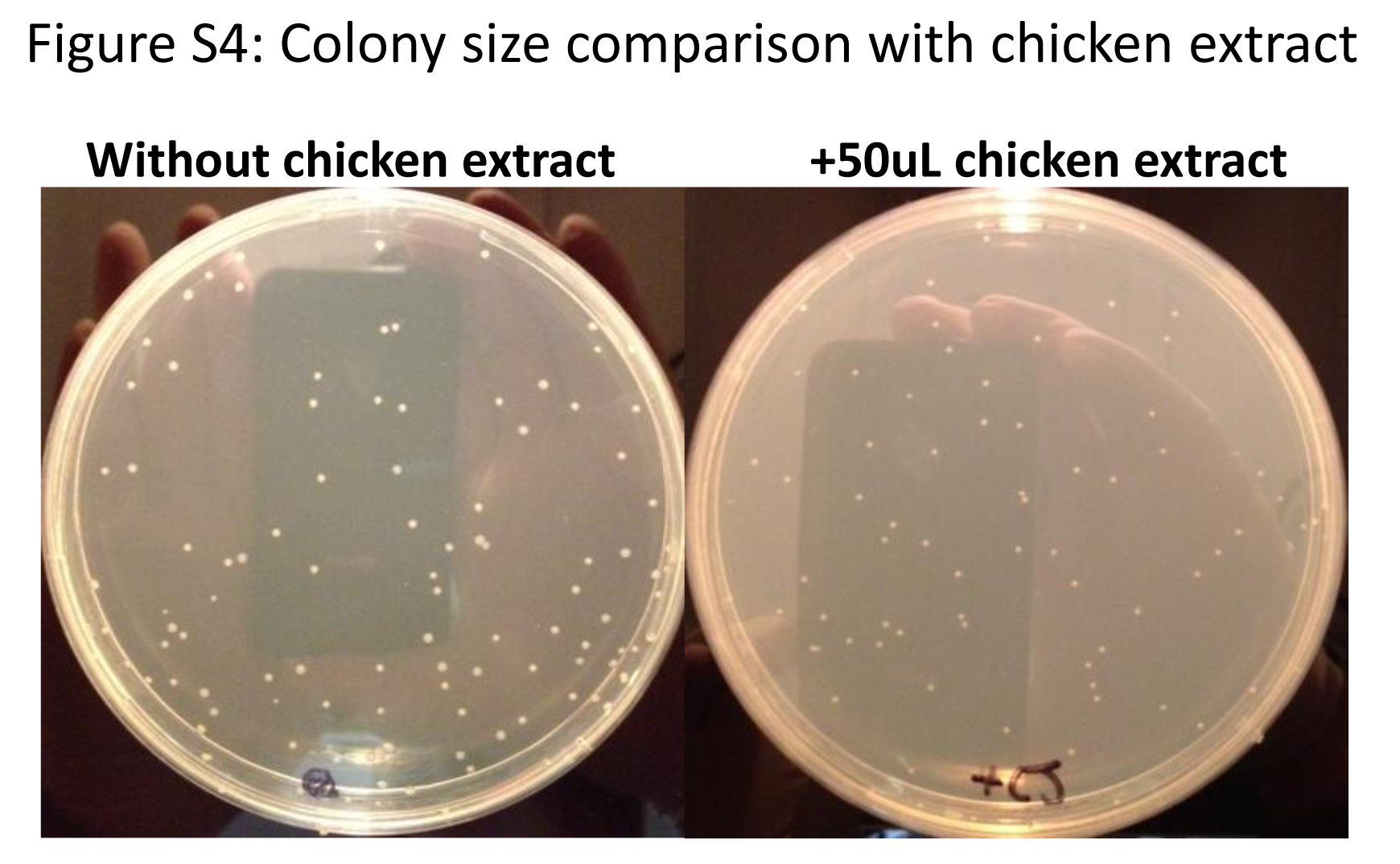

Supplement: Figure S4 — The effect of chicken extract on colony size. Adding 50 µL of chicken breast extract slows the growth of the Stx2f-expressing strain and the FSIS EC465-97 fluorescent control strain. The colonies on these plates are derived from the same two strain mixture as in Figure 5B. (TIF) [file pone.0076563.s004.tif]
